# 

**Published:** 1963-01

**Authors:** 


### Contents

| | | |
|---|---|---|
| EDITORIAL | | 1 |
| UNORTHODOXY IN MEDICINE AND RELIGION | *A. M. Maclachlan* | 2 |
| CANCER IN AFRICA | *J. N. P. Davies, M.D. (Bristol)* | 12 |
| DIRECT MEASUREMENT OF NOISE LEVELS IN A LARGE HOSPITAL | *A. P. Cole and P. H. Dimambro* | 18 |
| "TWO CAUSES FOR MACROCYTIC ANAEMIA IN ONE PATIENT" | *Clinico-Pathological Conference* | 22 |
| REPORTS FROM SOCIETIES | | 30 |
| NEWS FROM SOCIETIES | | 31 |

